# Supplementary material for: Oregano powder reduces Streptococcus and increases SCFA concentration in a mixed bacterial culture assay
Source: PLoS One. 2019 Dec 10;14(12):e0216853. doi: 10.1371/journal.pone.0216853 (PMC6903721; doi:10.1371/journal.pone.0216853)
Supplement: S1 File — (PDF) [file pone.0216853.s001.pdf]

Oregano powder reduces *Streptococcus* and increases SCFA concentration in a mixed bacterial culture assay

Benjamin W Bauer[1], Sheeana Gangadaoo[1], Yadav Bajagai[1], Thi Thu Hao Van[2], Robert J Moore[2] Dragana Stanley[1,*]

[1]Institute for Future Farming Systems, Central Queensland University, Rockhampton, Queensland, Australia

[2]RMIT University, School of Science, Bundoora, Victoria 3083, Australia

[*]Corresponding author
E-mail: D.Stanley@cqu.edu.au

Supplementary Data

Contents:

Table A: Concentrations of vitamins and trace elements in enhanced LYHBHI media

Table B: Differentially abundant OTUs

Table C: Genera and OTUs Spearman correlated to acetic acid

Table D: Genera and OTUs Spearman correlated to butyric acid

Table E: OTUs Spearman correlated to carvacrol

Table A: Concentrations of vitamins and trace elements in enhanced LYHBHI media.

Ingredient	Origin	Conc μg/L
Calcium (carbonate)	Cenovis Multivitamin	2.10E+01
Riboflavine (Vit B2)	Cenovis Multivitamin	4.00E-01
Thiamine Nitrate (B1)	Cenovis Multivitamin	4.00E-01
Cyanocobalamin (B12)	Cenovis Multivitamin	5.00E-03
Pyridoxine Hydrochloride (B6)	Cenovis Multivitamin	8.00E-01
Nicotinamide (B3)	Cenovis Multivitamin	4.00E+00
Calcium Panthonenate (B5)	Cenovis Multivitamin	2.20E+00
Zinc (Oxide)	Cenovis Multivitamin	1.50E+00
Ascorbic acid	Cenovis Multivitamin	4.50E+00
Cod-liver oil	Cenovis Multivitamin	1.75E+01
Magnesium (oxide heavy)	Cenovis Multivitamin	1.50E+00
Total Vit A	Cenovis Multivitamin	5.93E-02
dl-alpha-Tocopherol (Vit E 20IU)	Cenovis Multivitamin	1.82E+00
Iron (Ferrous Fumarate)	Cenovis Multivitamin	5.00E-01
Folic acid	Cenovis Multivitamin	3.00E-02
Betacarotene	Cenovis Multivitamin	1.20E-01
Cholecalciferol (Vit D3 154IU)	Cenovis Multivitamin	3.85E-04
Citrus Bioflavonoids Extract	Cenovis Multivitamin	2.00E-01
Biotin (Vit H)	Cenovis Multivitamin	1.50E-02
Phytomenadine (Vit K1)	Cenovis Multivitamin	1.50E-03
Iodine (Potassium Iodide)	Cenovis Multivitamin	1.50E-02

Copper (Cupric Sulfate Anhydrous)	Cenovis Multivitamin	1.00E-01
Chromium (Picolinate)	Cenovis Multivitamin	2.50E-03
Manganese (Sulfate Monohydrate)	Cenovis Multivitamin	1.00E-01
Selenium (Selenomethionine)	Cenovis Multivitamin	2.50E-03
Boron (Boric acid)	Cenovis Multivitamin	3.00E-01
Menaquinone 7-MK7	Caruso's	9.00E+00

Table B: Differentially abundant OTUs.

Positive Fold Change indicates fold higher in oregano, negative fold change represents fold reduced in oregano.

Multiple genera hits text in the table indicate blast hits with species from different genera with the same %ID, impossible to resolve on the current amplicon size.

Multiple sp. hits text in the table indicate blast hits with different species within the same genera that have the same %ID, impossible to resolve on the current amplicon size.

Uncult. bact. clone text in the table indicates number of hits to uncultured 16S bacterial gene sequences.

**OTUs (GreenGenes taxonomy)**	***p*-val (DESeq2)**	**Blastn hit**	**Blast % ID**	**Fold Change**
***Lactobacillus* OTU 152229**	2.70E-05	*L. ingluviei*	100	34.14
***Lactobacillus* OTU 272449**	3.20E-05	*L. acidophilus*	100	12.3
***Lactobacillus* OTU 104484**	6.10E-05	*L. ingluviei*	100	113100
***Lactobacillus* OTU 119821**	1.10E-04	*L. johnsonii*	100	8.123
**Lactobacillales OTU 33472**	3.40E-04	Multiple genera hits	100	6.034
***Lactobacillus* OTU 155425**	4.90E-04	*L. crispatus*	100	5.313
***Lactobacillus* OTU 27332**	9.70E-04	Uncult. bact. clone	98	73.79
***Streptococcus* OTU 207140**	1.00E-03	Uncult. bact. clone	99	-6.339
***Pediococcus* OTU 284103**	1.10E-03	Uncult. bact. clone	98	310100
***Lactobacillus* OTU 251983**	1.20E-03	Uncult. bact. clone	98	-6.113
***Lactobacillus* OTU 266421**	1.30E-03	Uncult. bact. clone	97	7.378

OTUs (GreenGenes taxonomy)	*p*-val (DESeq2)	Blastn hit	Blast % ID	Fold Change
***Lactobacillus* OTU 170328**	2.00E-03	*L. ingluviei*	96	6.261
**Lactobacillaceae OTU 101791**	2.40E-03	Multiple genera hits	99	159300
***Lactobacillus* OTU 17603**	2.70E-03	*L. ingluviei*	100	5.941
***Lactobacillus* OTU 80381**	3.50E-03	Uncult. bact. clone	97	17.24
***Lactobacillus* OTU 85290**	4.00E-03	*L. reuteri*	97	-9.651
**Enterobacteriaceae OTU 263876**	4.30E-03	Uncult. bact. clone	95	7.036
***Lactobacillus* OTU 169080**	5.20E-03	Uncult. bact. clone	95	29700
***Lactobacillus* OTU 76087**	5.20E-03	Multiple genera hits	99	6.504
***Lactobacillus* OTU 116794**	6.10E-03	Uncult. bact. clone	97	26820
***Streptococcus* OTU 136500**	6.20E-03	Uncult. bact. clone	97	-16.13
**Carnobacteriaceae OTU 9724**	6.70E-03	Multiple genera hits	98	4.884
***Streptococcus* OTU 167252**	6.80E-03	Uncult. bact. clone	98	-8.999
***Lactobacillus* OTU 213664**	7.40E-03	Uncult. bact. clone	98	9.258
**Streptococcaceae OTU 180394**	7.60E-03	Uncult. bact. clone	96	30660
***Lactobacillus* OTU 98212**	8.50E-03	Uncult. bact. clone	96	36270
***Enterococcus* OTU 123391**	9.20E-03	Multiple genera hits	95	21240
***Streptococcus* OTU 214315**	9.20E-03	Uncult. bact. clone	98	-5.219
**Streptococcaceae OTU 19535**	9.80E-03	*S. anginosus*	97	5.146
**Lactobacillaceae OTU 21754**	0.010	Uncult. bact. clone	97	55700
***Lactobacillus* OTU 211163**	0.010	Uncult. bact. clone	97	3.497
***Lactobacillus* OTU 238976**	0.010	Uncult. bact. clone	97	-16.66
**Lactobacillaceae OTU 186446**	0.011	Multiple genera hits	98	52220
***L. manihotivorans* OTU 159388**	0.011	Uncult. bact. clone	97	4.376
***Lactobacillus* OTU 300049**	0.011	Uncult. bact. clone	98	113.9
**Streptococcaceae OTU 182884**	0.012	Uncult. bact. clone	96	9.836
***L. coleohominis* OTU 224518**	0.013	*L. coleohominis*	100	2.939

**OTUs (GreenGenes taxonomy)**	***p*-val (DESeq2)**	**Blastn hit**	**Blast % ID**	**Fold Change**
***Lactobacillus* OTU 228314**	0.013	Uncult. bact. clone	96	10.44
***Lactobacillus* OTU 25404**	0.013	Multiple genera hits	96	4.216
***Lactobacillus* OTU 225126**	0.014	*L.* KC45b	97	44720
***Lactobacillus* OTU 88490**	0.014	*L. ingluviei*	97	12.64
***Lactobacillus* OTU 71614**	0.015	Uncult. bact. clone	96	8.038
***Streptococcus* OTU 97924**	0.015	Uncult. bact. clone	98	-6.752
***Lactobacillus* OTU 286390**	0.016	Uncult. bact. clone	97	20270
***Lactobacillus* OTU 708**	0.016	*L. ingluviei*	97	8.157
***Lactobacillus* OTU 123426**	0.017	Uncult. bact. clone	97	-20.05
***Enterococcus* OTU 38024**	0.019	Uncult. bact. clone	97	2.222
***Lactobacillus* OTU 61159**	0.019	*L. ingluviei*	96	21750
***Lactobacillus* OTU 145258**	0.021	Uncult. bact. clone	97	6.821
***Lactobacillus* OTU 250410**	0.021	Uncult. bact. clone	99	11.34
***Streptococcus* OTU 159558**	0.021	Uncult. bact. clone	97	-5.26
***Lactobacillus* OTU 192829**	0.023	Uncult. bact. clone	99	-4.384
***Lactobacillus* OTU 86477**	0.024	Uncult. bact. clone	98	-8.162
***Lactobacillus* OTU 151770**	0.025	*L. ingluviei*	97	2.977
***Lactobacillus* OTU 158017**	0.025	Uncult. bact. clone	95	31060
***Lactobacillus* OTU 269761**	0.025	Uncult. bact. clone	96	30260
***Streptococcus* OTU 106153**	0.025	*S. macedonicus*	100	-3.892
**Lactobacillaceae OTU 147775**	0.026	Uncult. bact. clone	96	29460
**Lactobacillales OTU 278065**	0.026	Uncult. bact. clone	98	44.97
***Lactobacillus* OTU 174211**	0.027	*L. ingluviei*	97	16530
***Streptococcus* OTU 82525**	0.027	Uncult. bact. clone	97	-5.678
***Lactobacillus agilis* OTU 261200**	0.028	*L. agilis*	99	-5.439
***Lactobacillus* OTU 56616**	0.028	Uncult. bact. clone	99	3.656
***Streptococcus* OTU 237114**	0.029	Uncult. bact. clone	97	-8.107
***Lactobacillus* OTU 109663**	0.030	Uncult. bact. clone	98	6.144
***Lactobacillus* OTU 258257**	0.030	Uncult. bact. clone	99	-20030

OTUs<br>(GreenGenes taxonomy)	*p*-val<br>(DESeq2)	Blastn hit	Blast<br>% ID	Fold<br>Change
***Lactobacillus* OTU 275395**	0.030	Uncult. bact. clone	97	6.727
***Streptococcus* OTU 144009**	0.030	Uncult. bact. clone	98	-12.45
***Streptococcus* OTU 61448**	0.030	Uncult. bact. clone	98	-26380
***Enterococcus* OTU 187731**	0.031	Uncult. bact. clone	98	2.284
***Lactobacillus* OTU 174201**	0.032	Uncult. bact. clone	98	2.526
***Lactobacillus* OTU 207893**	0.033	Uncult. bact. clone	98	-24070
***Lactobacillus* OTU 52161**	0.033	Uncult. bact. clone	98	2.42
***Lactobacillus* OTU 60926**	0.033	Uncult. bact. clone	98	12780
**Enterobacteriaceae OTU 92136**	0.033	Uncult. bact. clone	96	4.642
***Streptococcus* OTU 264005**	0.037	Uncult. bact. clone	99	-6.379
***Lactobacillus* OTU 192558**	0.038	Uncult. bact. clone	96	6.253
***Streptococcus* OTU 4154**	0.038	Uncult. bact. clone	97	-4.859
***Lactobacillus* OTU 184772**	0.039	Uncult. bact. clone	92	11220
***Enterococcus* OTU 175058**	0.040	Multiple genera hits	99	11420
***Lactobacillus* OTU 34587**	0.040	Uncult. bact. clone	99	4.445
***Lactobacillus* OTU 136182**	0.041	Multiple sp. hits (*Lactobacillus*)	97	4.301
***Lactobacillus* OTU 263116**	0.041	*L. reuteri*	97	-4.942
***Enterococcus* OTU 170058**	0.042	Multiple genera hits	96	5.834
***Lactobacillus* OTU 154837**	0.042	Uncult. bact. clone	96	11030
***Streptococcus* OTU 293956**	0.042	Uncult. bact. clone	98	-5.861
**Enterobacteriaceae OTU 253114**	0.042	Multiple sp. hits (*Enterobacteriaceae*)	97	13740
***Lactobacillus* OTU 226170**	0.043	Uncult. bact. clone	99	2.021
***Lactobacillus* OTU 207731**	0.044	Uncult. bact. clone	99	10390
***Streptococcus* OTU 122385**	0.044	Uncult. bact. clone	96	-4.077
***Lactobacillus* OTU 284267**	0.045	Uncult. bact. clone	95	7.555
***Lactobacillus* OTU 126939**	0.046	Uncult. bact. clone	97	5.421
***Lactobacillus* OTU 3668**	0.046	*L. ingluviei*	97	13770

**OTUs (GreenGenes taxonomy)**	***p*-val (DESeq2)**	**Blastn hit**	**Blast % ID**	**Fold Change**
***L. agilis* OTU 116383**	0.048	*L. agilis*	96	11300
***Lactobacillus* OTU 9908**	0.048	Uncult. bact. clone	95	20080
***Lactobacillus* OTU 264260**	0.049	Uncult. bact. clone	96	-15460

Table C: Genera and OTUs Spearman correlated to acetic acid.

Significant correlations for top 100 most abundant OTUs are shown.

Multiple genera hits text in the table indicate blast hits with species from different genera with the same %ID, impossible to resolve on the current amplicon size.

Uncult. bact. clone text in the table indicates number of hits to uncultured 16S bacterial gene sequences.

**OTUs (GreenGenes taxonomy)**	***p*-val**	**R**	**Blastn hit**	**Blast % ID**
**Enterobacteriaceae OTU 271191**	1.70E-04	0.88	Uncult. bact. clone	96
**Enterobacteriaceae OTU 261461**	5.00E-04	0.85	Uncult. bact. clone	97
***Lactobacillus* OTU 200355**	6.30E-04	0.84	Uncult. bact. clone	98
***Enterococcus* OTU 15749**	1.00E-03	0.82	*E. faecium*	100
***Enterococcus* OTU 55837**	1.70E-03	0.80	Uncult. bact. clone	98
***Enterococcus* OTU 11689**	1.80E-03	0.80	Uncult. bact. clone	97
**Enterobacteriaceae OTU 267130**	3.40E-03	0.77	Uncult. bact. clone	99
***Enterococcus* OTU 187731**	4.70E-03	0.75	Uncult. bact. clone	98
***Lactobacillus* OTU 17603**	5.30E-03	-0.75	*L. ingluviei*	100
**Enterobacteriaceae OTU 146685**	5.30E-03	0.75	Uncult. bact. clone	98
**Enterobacteriaceae OTU 224864**	6.60E-03	0.73	Uncult. bact. clone	99
***Lactobacillus* OTU 35668**	0.010	-0.71	*L. marseille*	98
**Enterobacteriaceae OTU 135153**	0.011	0.70	*E. coli*	100
***Lactobacillus* OTU 203797**	0.014	-0.69	Uncult. bact. clone	96
***Streptococcus* OTU 154406**	0.014	-0.68	Uncult. bact. clone	98
***Lactobacillus* OTU 708**	0.015	-0.68	*L. ingluviei*	97
***Lactobacillus* OTU 109663**	0.020	-0.66	Uncult. bact. clone	98
***Enterococcus* OTU 264003**	0.021	0.66	Uncult. bact. clone	98
***L. manihotivorans* OTU 159388**	0.021	-0.66	Uncult. bact. clone	97
**Enterobacteriaceae OTU 183643**	0.023	0.65	Uncult. bact. clone	100
***Lactobacillus* OTU 76947**	0.024	0.64	Uncult. bact. clone	97
***S. alactolyticus* OTU 147828**	0.024	0.64	Uncult. bact. clone	99

**OTUs (GreenGenes taxonomy)**	***p*-val**	**R**	**Blastn hit**	**Blast % ID**
***Lactobacillus* OTU 148643**	0.025	-0.64	*L. johnsonii*	100
***L. manihotivorans* OTU 198669**	0.026	0.64	Uncult. bact. clone	98
***Lactobacillus* OTU 151770**	0.027	-0.63	*L. ingluviei*	97
***Lactobacillus* OTU 52161**	0.035	0.61	Uncult. bact. clone	98
***Lactobacillus* OTU 92052**	0.035	-0.61	Uncult. bact. clone	99
***Lactobacillus* OTU 1550**	0.038	-0.60	*L. KC45b*	97
**Streptococcaceae OTU 233731**	0.040	-0.60	Uncult. bact. clone	99
***L. coleohominis* OTU 224518**	0.040	-0.60	Uncult. bact. clone	100
***Lactobacillus* OTU 287135**	0.041	-0.60	Uncult. bact. clone	100
***Lactobacillus* OTU 44522**	0.042	-0.59	Uncult. bact. clone	99
***Lactobacillus* OTU 1270**	0.050	-0.58	Uncult. bact. clone	98
**GENERA**				
Enterococcus	2.20E-04	0.87	-	-
Ruminococcus	4.00E-04	0.85	-	-
**Unclassified Coriobacteriaceae**	8.20E-04	0.83	-	-
**Unclassified Burkholderiales**	4.50E-03	0.75	-	-
Proteus	5.50E-03	0.74	-	-
**Unclassified Betaproteobacteria**	5.70E-03	0.74	-	-
Coprobacillus	8.50E-03	0.72	-	-
Bacteroides	9.60E-03	0.71	-	-
**Unclassified Enterobacteriaceae**	0.011	0.70	-	-
Sutterella	0.012	0.69	-	-
Clostridium	0.015	-0.68	-	-
**Unclassified Enterococcaceae**	0.017	0.67	-	-
**Unclassified Erysipelotrichaceae**	0.019	0.66	-	-
Bifidobacterium	0.025	0.64	-	-
Collinsella	0.028	0.63	-	-
**Unclassified Ruminococcaceae**	0.032	0.62	-	-
**Unclassified Pseudomonadaceae**	0.040	0.60	-	-

Table D: Genera and OTUs Spearman correlated to butyric acid.

Significant correlations for top 100 most abundant OTUs are shown.

Multiple genera hits text in the table indicate blast hits with species from different genera with the same %ID, impossible to resolve on the current amplicon size.

Uncult. bact. clone text in the table indicates number of hits to uncultured 16S bacterial gene sequences.

**OTUs (GreenGenes taxonomy)**	***p*-val**	**R**	**Blastn hit**	**Blast % ID**
**Enterobacteriaceae OTU 261461**	3.20E-04	0.86	Uncult. bact. clone	99
**Enterobacteriaceae OTU 271191**	5.80E-04	0.84	Uncult. bact. clone	96
***Lactobacillus* OTU 200355**	1.10E-03	0.82	Uncult. bact. clone	98
***Enterococcus* OTU 55837**	1.30E-03	0.81	Uncult. bact. clone	98
***Enterococcus* OTU 187731**	1.60E-03	0.80	Uncult. bact. clone	98
***Enterococcus* OTU 15749**	1.80E-03	0.80	*E. Faecium*	100
***Enterococcus* OTU 11689**	4.30E-03	0.76	Uncult. bact. clone	98
**Enterobacteriaceae OTU 267130**	6.30E-03	0.74	Uncult. bact. clone	99
***Lactobacillus* OTU 76947**	6.90E-03	0.73	Uncult. bact. clone	95
**Enterobacteriaceae OTU 146685**	7.00E-03	0.73	Uncult. bact. clone	98
***Lactobacillus* OTU 17603**	9.00E-03	-0.71	*L. ingluviei*	100
**Enterobacteriaceae OTU 224864**	0.011	0.70	Uncult. bact. clone	99
***Enterococcus* OTU 264003**	0.012	0.70	Uncult. bact. clone	98
**Enterobacteriaceae OTU 135153**	0.014	0.69	*E. coli*	99
***Lactobacillus* OTU 35668**	0.015	-0.68	Multiple sp. hits (*Lactobacillus*)	98
***S. alactolyticus* OTU 147828**	0.018	0.67	Uncult. bact. clone	99
***Lactobacillus* OTU 109663**	0.021	-0.65	Uncult. bact. clone	99
**Enterobacteriaceae OTU 183643**	0.021	0.65	Uncult. bact. clone	98
***L. manihotivorans* OTU 198669**	0.028	0.63	Uncult. bact. clone	98
***Lactobacillus* OTU 52161**	0.028	0.63	Uncult. bact. clone	98
***Lactobacillus* OTU 203797**	0.034	-0.61	Uncult. bact. clone	96
***Lactobacillus* OTU 151770**	0.034	-0.61	*L. ingluviei* JCM 12531	97

**OTUs**<br>**(GreenGenes taxonomy)**	***p*-val**	**R**	**Blastn hit**	**Blast**<br>**% ID**
***Enterococcus* OTU 93226**	0.035	0.61	*L. ingluviei*	97
***Lactobacillus* OTU 708**	0.036	-0.61	Uncult. bact. clone	97
***L. manihotivorans* OTU 159388**	0.038	-0.60	Multiple genera hits	100
***Lactobacillus* OTU 148643**	0.040	-0.60	Uncult. bact. clone	97
**Streptococcaceae OTU 233731**	0.041	-0.60	Uncult. bact. clone	99
***Lactobacillus* OTU 92052**	0.043	-0.59	Uncult. bact. clone	99
***Lactobacillus* OTU 66484**	0.044	0.59	Uncult. bact. clone	97
***Lactobacillus* OTU 1550**	0.046	-0.58	*L. KC45b*	97
***Lactobacillus* OTU 44522**	0.046	-0.59	Uncult. bact. clone	99
**GENERA**				
Enterococcus	5.00E-04	0.85	-	-
Ruminococcus	1.50E-03	0.81	-	-
**Unclassified Coriobacteriaceae**	2.70E-03	0.78	-	-
**Unclassified Burkholderiales**	5.20E-03	0.75	-	-
Proteus	5.90E-03	0.74	-	-
Coprobacillus	6.50E-03	0.73	-	-
Sutterella	6.80E-03	0.73	-	-
Bacteroides	0.012	0.69	-	-
**Unclassified Betaproteobacteria**	0.013	0.69	-	-
**Unclassified Enterobacteriaceae**	0.014	0.69	-	-
Clostridium	0.017	-0.67	-	-
**Unclassified Enterococcaceae**	0.023	0.65	-	-
**Unclassified Ruminococcaceae**	0.032	0.62	-	-

Table E: OTUs Spearman correlated to carvacrol.

Significant correlations for top 100 most abundant OTUs are shown. There were no genera significantly correlated with carvacrol.

Multiple genera hits text in the table indicate blast hits with species from different genera with the same %ID, impossible to resolve on the current amplicon size.

Uncult. bact. clone text in the table indicates number of hits to uncultured 16S bacterial gene sequences.

**OTUs (GreenGenes taxonomy)**	***p*-val**	**R**	**Blastn hit**	**Blast % ID**
***Lactobacillus* OTU 192829**	6.30E-04	-0.84	Uncult. bact. clone	99
***Lactobacillus* OTU 152229**	1.20E-03	0.82	Uncult. bact. clone	97
***Streptococcus* OTU 214315**	1.60E-03	-0.80	Uncult. bact. clone	98
***Lactobacillus* OTU 251983**	5.20E-03	-0.75	Uncult. bact. clone	98
***Streptococcus* OTU 207140**	6.40E-03	-0.74	Uncult. bact. clone	99
***Streptococcus* OTU 230511**	0.011	-0.70	Uncult. bact. clone	99
***L. manihotivorans* OTU 159388**	0.012	0.69	Uncult. bact. clone	97
***Lactobacillus* OTU 119821**	0.014	0.68	*L. KC38*	99
***Lactobacillus* OTU 85290**	0.016	-0.67	Uncult. bact. clone	97
***Streptococcus* OTU 52564**	0.019	-0.66	Uncult. bact. clone	98
**Lactobacillales OTU 33472**	0.019	0.66	Uncult. bact. clone	99
***Streptococcus* OTU 122385**	0.023	-0.65	Uncult. bact. clone	96
***Lactobacillus* OTU 272449**	0.026	0.64	Uncult. bact. clone	99
***Streptococcus* OTU 159558**	0.035	-0.61	Uncult. bact. clone	99
***Lactobacillus* OTU 92052**	0.038	0.60	Uncult. bact. clone	99
***Lactobacillus* OTU 1550**	0.039	0.60	*L. KC45b*	97
